# Hybrid extrachromosomal DNA in HPV-driven cancers

**DOI:** 10.1128/jvi.01689-25

**Published:** 2026-06-30

**Authors:** Benjamin Hafey, Liangqi Xie

**Affiliations:** 1 Microbial Sciences in Health, Cleveland Clinic Research, Cleveland Clinic 2569, Cleveland, Ohio, USA; 2 Molecular Medicine, Cleveland Clinic Lerner College of Medicine of Case Western Reserve University School of Medicine, Cleveland Clinic 2569, Cleveland, Ohio, USA; 3 Case Comprehensive Cancer Center 151230, Cleveland, Ohio, USA; Universiteit Gent, Merelbeke, Belgium

**Keywords:** extrachromosomal DNA, episome, hybrid, HPV

## Abstract

Extrachromosomal DNA (ecDNA) drives extensive oncogene amplification and intrinsic tumor heterogeneity in human cancers. Recent studies have uncovered an unexpected convergence between viral oncogenesis and ecDNA biology: integration of human papillomavirus (HPV) can trigger the formation of hybrid viral-human ecDNA structures in approximately 30% of HPV-associated oropharyngeal cancers (HPVOPC), with analogous structures reported in cervical cancer models. These chimeric circular elements fuse viral oncogenes with captured human genomic sequences, co-amplifying viral and human genes, and generating *de novo* enhancer complexes absent from either parental structure. Hybrid ecDNA exhibits canonical ecDNA features, including disproportionately high gene expression, accessible chromatin landscape, and the capacity to act as mobile trans-activating elements that potentiate oncogenic programs. Emerging evidence further suggests that hybrid ecDNA operates through distinct regulatory mechanisms and may create specific therapeutic vulnerabilities that can be leveraged for personalized cancer therapy. Elucidation of the mechanisms governing hybrid ecDNA biogenesis, maintenance, and function may therefore uncover exploitable dependencies in hybrid ecDNA-associated cancers. In this review, we synthesize current insights into hybrid ecDNA formation, regulatory mechanisms, and therapeutic opportunities and discuss how these advances may guide the development of precision cancer therapies.

## INTRODUCTION

Circular, extrachromosomal DNA (ecDNA) has emerged as a hallmark of human cancers (1). These cancer-associated ecDNAs have been studied in parallel with another class of circular DNA elements—oncogenic viral episomes. Recent studies have revealed an unexpected convergence between these two fields, revealing hybrid viral-human ecDNA structures in HPV-associated cancers (2–6). We discuss how these viral-human chimeric structures blur traditional boundaries between virology and cancer biology, and how a mechanistic understanding of this convergence could inform the development of new therapies for hybrid ecDNA-positive malignancies. As the field remains nascent, we also delineate key open questions surrounding hybrid ecDNA biogenesis, maintenance, and evolutionary significance that must be addressed to fully realize this therapeutic potential.

## TWO PARADIGMS OF EXTRACHROMOSOMAL DNA AMPLIFICATION

The discovery of ecDNA has transformed our understanding of cancer biology (1, 7–10). These circular, often megabase-scale elements amplify oncogenes across the vast majority of human cancer types (25 of 29 analyzed) (11); however, they are largely absent from normal tissues, underscoring their cancer-specific nature (10, 11). Although the mechanisms of ecDNA biogenesis remain incompletely understood, ecDNA is thought to arise from chromosomal instability through processes such as breakage-fusion-bridge cycles (11, 12), chromothripsis (12, 13), and other events that excise and circularize genomic elements (12, 14). Large-scale cancer genomic analyses further demonstrated that ecDNA frequency increases from primary tumors to advanced metastatic disease, consistent with a selective advantage during tumor progression (15). Moreover, the presence of ecDNA in cancer patients is associated with significantly worse 5-year survival compared to those who harbored non-circular or no amplifications (11). Together, the profound clinical relevance of this extrachromosomal phenomenon, together with fundamental questions it raises regarding cancer evolution and therapeutic resistance, led to the designation of ecDNA as a Cancer Grand Challenge in 2020 (16).

The poor prognosis associated with ecDNA can be attributed to at least two intrinsic properties. First, ecDNA drives massive oncogene transcription: ecDNA-encoded oncogenes consistently rank among the most highly expressed genes in cancer (1, 10, 17, 18), even after normalizing for copy number (17). This excessive oncogene transcription is mediated in part by hyperaccessible chromatin structure (17), enhancer hijacking (19), and cooperative intermolecular interactions within three-dimensional ecDNA hubs (18, 20), among other mechanisms (21). Second, ecDNA exhibits non-Mendelian inheritance. Because ecDNA lack centromeres, they rely on tethering to mitotic chromosomes for nuclear inheritance, resulting in uneven segregation among daughter cells that promotes intratumoral heterogeneity and accelerates the emergence of therapeutic resistance (1, 10, 12, 22–24).

In addition to ecDNA, oncogenic viral episomes represent another class of extrachromosomal circular DNA in cancer. Among seven oncogenic viruses that contribute to approximately 15%–20% of human cancers worldwide (25, 26), five DNA tumor viruses, HPV, hepatitis B virus (HBV), Epstein-Barr virus (EBV), Kaposi’s sarcoma-associated herpesvirus (KSHV), and Merkel cell polyomavirus (MCPyV), can persist as episomes that share key structural and functional features with ecDNA (Table 1), including a circular double-stranded DNA structure and extrachromosomal maintenance (27). Some episomes (e.g., HPV, EBV, and KSHV) can tether to host mitotic chromosomes for nuclear retention (28, 29), and the EBV episome can engage in long-range physical interactions with chromosomal regulatory elements (30–32), a capacity that parallels the trans-regulatory activity described for ecDNA (33, 34).

**TABLE 1:** Comparison of conventional extrachromosomal DNA and viral episomes^*a*^

Feature	Conventional ecDNA	Oncogenic viral episomes
Structure	Circular, extrachromosomal, no centromere (1, 7–10)	Circular, extrachromosomal, no centromere (27, 35)
Content	Human oncogenes + regulatory elements (1, 8, 10)	Viral genome (27, 35)
Primary oncogenic driver	Massive oncogene amplification (MYC, EGFR) (1, 8, 10)	Viral oncoproteins (E6/E7 for HPV, EBNA1 for EBV, and LANA for KSHV) (35)
Size	Hundreds of kbp to several Mbp (1, 8, 36)	~8 kbp (HPV) (37), ~170 kbp (EBV [27] and KSHV [38, 39])
Copy number	Tens to hundreds (10)	Varies by phase of infection (EBV [40, 41], HPV [42], KSHV [43])
Gene expression	Very high (ranked top within cancer cells) (1, 10, 17)	Context dependent (35)
Replication	Host machinery (mechanisms unclear)	Viral origin, viral proteins, and host machinery (28, 35, 44)
Mitotic inheritance	Host protein (BRD4) promoted RNA scaffolding (22)	Episomal maintenance proteins (E2 for HPV, EBNA1 for EBV, and LANA for KSHV) (28, 29)
Chromatin state and organization	Hyperaccessible (1, 8, 17)	CTCF-cohesin-mediated chromatin loops (HPV [45, 46], EBV [45, 47, 48], and KSHV [45, 49])
Regulatory elements	Cellular enhancers (hijacked or translocated) (21)	Viral regulatory elements, including origins of replication, promoters, and enhancer regions (28, 50, 51)
Trans-regulation	Yes; mobile enhancers activate chromosomal genes (33, 34)	Yes (32)
Formation model/mechanism	Chromothripsis (12, 13) and breakage-fusion-bridge (BFB) cycles (12, 14)	Viral infection and episomal replication (28, 37)

^*a*^ EGFR, epidermal growth factor receptor; BRD4, bromodomain-containing protein 4; HPV, human papillomavirus; EBNA1, Epstein-Barr nuclear antigen 1; EBV, Epstein-Barr virus; LANA, latency-associated nuclear antigen; KSHV, Kaposi's sarcoma-associated herpesvirus; CTCF, CCCTC-binding factor.

Despite these similarities, important distinctions exist between viral episomes and ecDNA (Table 1). Viral episomes encode dedicated episome maintenance proteins (28), such as HPV E2 (44), KSHV latency associated nuclear antigen (LANA) (52), and EBV nuclear antigen 1 (EBNA1), which coordinate transcriptional regulation, DNA replication, and mitotic segregation/partitioning. For example, the HPV E2 protein is essential for the episomal life cycle: it recruits the viral helicase E1 to initiate genome replication and forms a molecular bridge with the host bromodomain-containing protein 4 (BRD4), thereby tethering the viral episomes to mitotic chromosomes and ensuring their faithful segregation into daughter cells during cell division (44, 53–56). In contrast, ecDNAs are derived from the cancer genome and lack virus-encoded maintenance machinery; instead, they co-opt host cellular factors, including BRD4, to support transcriptional regulation (18, 22) and centromere-independent inheritance (22). These distinct maintenance strategies, along with others, have long defined viral episomes and ecDNA as separate entities in cancer biology. However, recent discoveries reveal an unexpected convergence: oncogenic viruses hijack the conventional ecDNA amplification pathway, generating hybrid structures that blur the boundaries between these two paradigms.

## HYBRID ecDNA

Recent analyses of HPV-associated cancers have revealed hybrid extrachromosomal structures that, unlike conventional ecDNA, incorporate both viral and human genomic sequences—a phenomenon observed thus far in HPVOPC and cervical cancer (2–6). HPV is the etiological agent for virtually all cervical cancers and the majority of oropharyngeal cancers (57, 58). While HPV-driven oncogenesis can involve mutations or epigenetic alterations (57, 58), viral genome integration—often disrupting *E2*, the viral transcriptional repressor of the oncogenes *E6* and *E7*, and frequently co-disrupting *E1*—is thought to be a major oncogenic pathway and is observed in ~71% of HPV-positive head and neck cancers and ~83% of HPV-positive cervical cancers (57–62). Notably, Pang et al. observed ~30% (16 out of 56) of analyzed HPVOPC cases harbor hybrid viral-human ecDNA structures (2). These ecDNA structures can coexist with chromosomal integration, representing alternative or complementary structural configurations that may further potentiate oncogene expression during oncogenesis (2, 5). The molecular mechanisms underlying hybrid ecDNA formation, maintenance, and transcriptional regulation create a distinct combination of features not seen in their predecessor HPV episomes.

### Proposed hybrid ecDNA biogenesis models

Hybrid ecDNA constitutes a true molecular chimera, combining viral oncogenes and regulatory elements with captured human genomic segments into a single circular DNA molecule, a configuration absent from either parent alone (2, 3, 5). Its formation appears a direct consequence of viral integration, as whole-genome sequencing of HPV-positive cancers revealed a strong association between viral integration and adjacent focal genomic instability (e.g., amplifications, deletions, inversions, and translocations) (63). To explain this, Akagi et al. proposed a “looping” model in which integrated HPV sequences bridge noncontiguous chromosomal sites and undergo rolling circle amplification, generating structured viral-host concatemers with recurrent breakpoints that retain *E6* and *E7*, a process distinct from the disorganized fragmentation characteristic of chromothripsis (63). Complementary long-read sequencing of HPV16-positive cervical cancer cell lines supports this model, revealing structurally diverse viral concatemers comprising full-length and deleted viral genomes in variable arrangements across multiple integration sites. These findings are consistent with prior extrachromosomal concatemer formation followed by chromosomal insertion, a process termed HPV “superspreading” (37).

**Fig 1:**
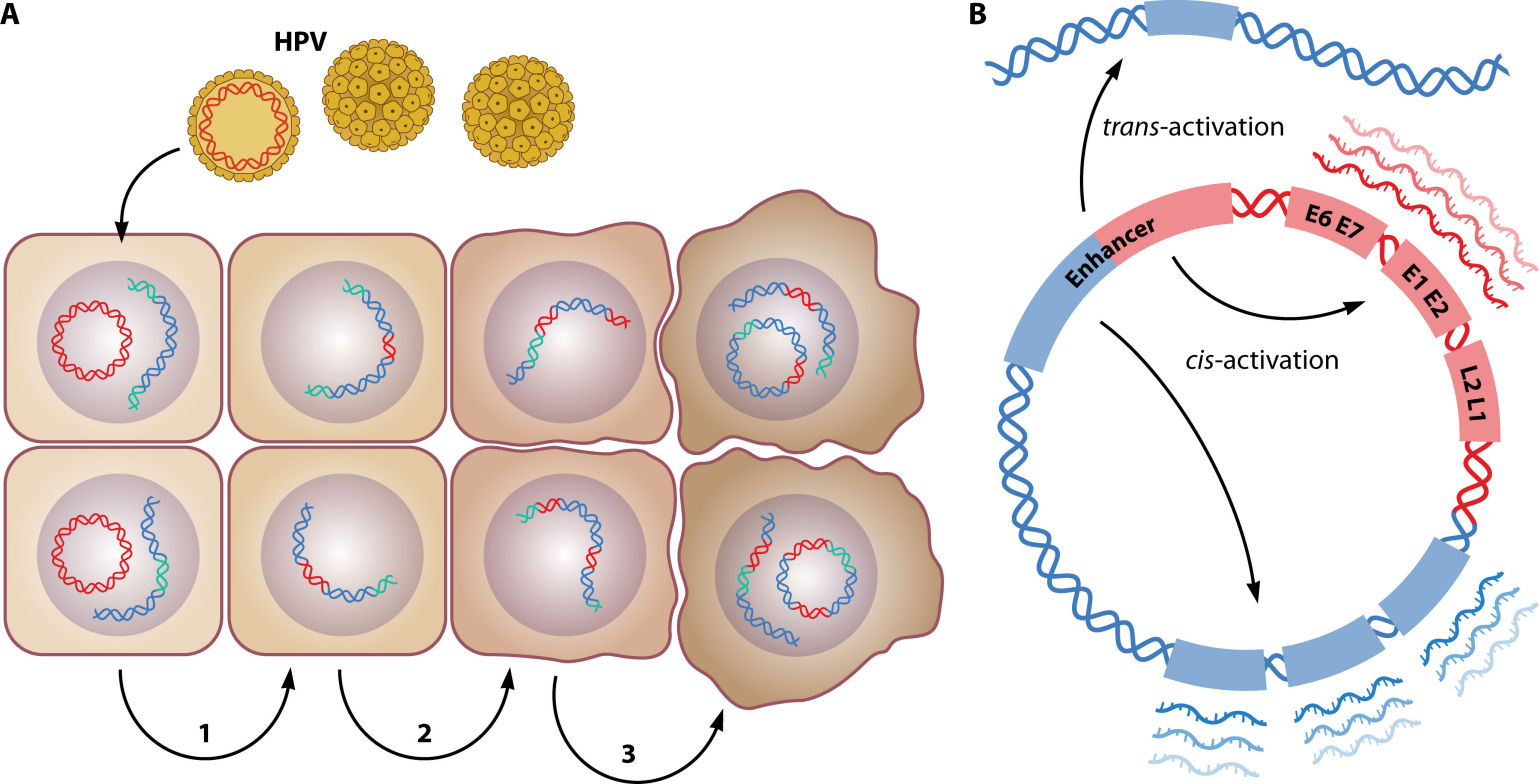
HPV infection initiates hybrid ecDNA formation that exhibits a unique regulatory landscape. (**A**) Simplified schematic of HPV heterocateny (adapted from reference (5), previously published under a CC BY-NC-ND 4.0 license) (1). Concatemerized HPV genomes (red) integrate into host chromosomes (blue) (2). Amplification and recombination generate diverse virus-host concatemers (3). Excision from host chromosomes captures flanking host DNA, producing diverse extrachromosomal and intrachromosomal virus-host DNA forms. In practice, steps 2 and 3 are iterative, and additional rounds of excision, amplification, and recombination continue to drive intratumoral heterogeneity and clonal evolution. (**B**) Hybrid ecDNA forms composite enhancers from juxtaposed viral and human regulatory elements. These enhancers activate genes both *in cis* (on the same ecDNA molecule) and *in trans* (on other ecDNA copies and chromosomal loci), promoting excessive expression of viral and human genes.

More recently, integrated whole-genome analysis of primary oropharyngeal tumors and cell lines using Illumina short-read sequencing and PacBio HiFi/Oxford Nanopore Technologies long-read sequencing led Akagi et al. to identify “heterocateny,” a previously undescribed form of genomic structural variation characterized by diverse, interrelated, and repetitive patterns of concatemerized virus and host DNA segments within a tumor (Fig. 1A) (5). In this model, unstable concatemerized HPV genomes are inserted into and excised from chromosomes, facilitating capture, amplification, and recombination of host DNA by rolling-circle replication, recombination-dependent replication, and homology-directed repair, producing structurally heterogeneous virus-host DNA rearrangements in both extrachromosomal and intrachromosomal forms that promote intratumoral heterogeneity and clonal evolution. This model is supported by independent studies: Zhou et al. identified shared integration breakpoints across distinct clonal integration events, HPV-mediated interchromosomal translocations, and circular virus-human hybrid DNA structures in HPV16-positive cervical tumors (6), and Porter et al. reported heterologous integrants with multiple distinct viral genome configurations in 21% of breakpoint pairs across 72 long-read sequenced primary cervical tumors, consistent with a heterocateny-like process (64).

### Regulatory mechanisms of hybrid ecDNA

Beyond structural heterogeneity, the chimeric viral-human composition endows hybrid ecDNA with distinctive regulatory capabilities absent from canonical viral episomes. Hybrid ecDNA varies widely in size, from <10 kbp to >100 kbp depending on the extent of captured human sequence (2, 4), and occurs at relatively low copy numbers (e.g., ~4/cell (3)) compared to conventional ecDNAs, which typically span Mbp (36) and accumulate at tens to hundreds of copies per cell (10). Despite this, hybrid ecDNA sustains robust viral oncogene expression (3). This observation aligns with findings from Nulton et al. that HPV copy number alone does not necessarily correlate with *E6/E7* expression (65). Instead, elevated oncogene expression likely arises from the unique regulatory properties of hybrid ecDNA, including open chromatin structure, *de novo* enhancer formation, coordinated gene co-amplification (Fig. 1B), and cooperative spatial clustering.

First, hybrid ecDNA harbors open chromatin architecture. Nakagawa et al. demonstrated that hybrid ecDNA is enriched for open chromatin regions and active enhancer marks (3), reminiscent of the hyperaccessible chromatin state characteristic of cancer-derived ecDNA that lacks canonical nucleosomal organization (17). This permissive chromatin environment likely underlies the ability of hybrid ecDNA to regulate heightened transcription.

Second, hybrid ecDNA can create *de novo* composite enhancer architectures that drive oncogene expression. Consistent with this model, Nakagawa et al. identified a composite enhancer architecture formed by juxtaposition of viral *L1* enhancer elements with captured human enhancers, jointly activating both viral (*E6/E7*) and human genes on the same hybrid ecDNA molecule (3). Similarly, Tian et al. reported that HPV integration generates *de novo* cellular enhancers that can be excised and embedded within hybrid ecDNA, enabling both intrachromosomal and interchromosomal interactions with target gene promoters (4).

Third, hybrid ecDNA co-amplifies oncogenic host genes alongside viral sequences, generating a coordinated viral-human transcriptional program with oncogenic potential. Pang et al. reported that 86.8% of host genes captured on hybrid ecDNA amplicons across HPVOPCs are oncogenes or immune evasion genes and are strongly overexpressed (mean Fragments Per Kilobase per Million or FPKM ratio 149.8, median 4.26, relative to tumors lacking the gene on ecDNA) (2). Notable examples included *TNFSF4* (116.6-fold), EGFR (30.7-fold), and *CD274* (24.4-fold) (2). Additional studies identified cancer driver genes such as *MYC* (5) and *NR4A2* (65) co-amplified on hybrid ecDNA structures. Pang *et al*. further observed transcript-level fusion formation (2), mirroring the canonical ecDNA behavior that contributes to oncogenic function and diversity (66). These studies support the notion that host gene capture and amplification on hybrid ecDNA may synergistically enhance oncogenic output.

Finally, hybrid ecDNA spatially clusters within the nucleus, creating the potential to utilize cooperative regulatory hubs. Beyond noticing this, Nakagawa et al. observed that pharmacological inhibition of BRD4 using the bromodomain and extra terminal domain (BET) family pan-inhibitor JQ1 results in a hybrid-ecDNA-specific reduction in *E6/E7* expression and tumor growth (3). These findings parallel the ecDNA “hub” model, in which BRD4 scaffolding promotes intermolecular ecDNA interactions and potentiates oncogene transcription (18), suggesting shared higher-order principles governing extrachromosomal gene regulation.

### Hybrid ecDNA: adaptive evolution or genomic accident?

Despite the apparent oncogenic potential as a result of amplifying the expression of diverse oncogenic and immune evasion genes (2, 5, 65), the evolutionary origin and selective advantage of hybrid ecDNA remain unresolved. Two non-mutually exclusive models may describe different phases of the same process.

First, the adaptive evolution model, consistent with clonal evolution theory (67) and sequential selective sweeps during tumorigenesis, posits that hybrid ecDNA is positively selected and enhances oncogenic fitness through coordinated viral-human gene expression. Evidence for this model begins at the level of HPV integration. Symer et al. identified recurrent HPV integration sites near oncogenic and immune evasion genes including *MYC*, *SOX2*, *TP63*, *FGFR3*, and *CD274* across 105 HPV-positive oropharyngeal cancers, attributing recurrence to clonal selection rather than directed integration. They proposed that initial integration occurs randomly in open chromatin regions, with subsequent clonal selection for growth advantages (68). Similar conclusions from cervical cancer studies (69, 70) support this two-step model in which largely stochastic integration is followed by selection of oncogenically favorable clones. Khan et al. identified recurrent *FGFR3-TACC3* oncogenic fusions arising from HPV concatemer integration followed by clonal selection (71). Nakagawa et al. further observed that HPV integration in hybrid ecDNA contexts occurs at apparently random genomic locations and can generate local enhancer activity, bypassing the requirement for a pre-existing host regulatory landscape (3). Functional studies further support a fitness advantage conferred by hybrid ecDNA. Pang et al. demonstrated that the intact chimeric transcript from co-amplified hybrid ecDNA significantly induced proliferation when overexpressed (e.g., co-amplified *TTC33* gene) or suppressed proliferation when knocked down (e.g., FOXE1) (2). CRISPR interference (CRISPRi) targeting of *de novo* enhancers, as well as BET inhibition, selectively suppressed *E6/E7* expression and tumor growth in hybrid ecDNA-positive models, indicating functional dependence on these structures rather than mere co-occurrence (3).

The second model, neutral emergence with latent selective potential, describes the earlier phase of this process, focusing on how hybrid ecDNA arises before selection (72, 73). In this view, viral integration generates DNA substrates permissive to hybrid ecDNA formation through stochastic genomic processes, producing structural diversity that is initially neutral with respect to fitness. Supporting this, hybrid ecDNA was detected in only ~30% of HPVOPC (2), indicating that its presence does not confer a uniform selective advantage. Moreover, the structural heterogeneity of virus-host concatemers across individual tumor cells, driven by dynamic cycles of chromosomal insertion, excision, and recombination, is consistent with a process driven by genomic instability (5). Additionally, the incorporation of HPV regulatory elements can sustain transcriptional autonomy (3, 4), potentially alleviating selective pressure for integration at specific genomic loci.

These two models are complementary rather than competing. The neutral model explains the structural diversity in hybrid ecDNA, whereas the adaptive model explains the selective enrichment of specific configurations within that diversity. Akagi et al. explicitly recognized this duality, noting that heterocateny simultaneously drives both genetic variation and clonal selection (5). Distinguishing the relative contributions of these phases will require analysis of larger patient cohorts that correlate hybrid ecDNA status with tumor fitness and clinical outcomes, quantitative estimation of selection coefficients in relevant cellular systems, and longitudinal analyses that resolve the temporal relationship of hybrid ecDNA emergence and clonal expansion.

### Research approaches for hybrid ecDNA

The characterization of hybrid ecDNA structures relies on complementary sequencing, computation, and imaging approaches. Many recent hybrid ecDNA studies (2–5) have employed Illumina short-read whole-genome sequencing to identify copy number alterations, structural variants, and virus-host breakpoints. However, the intrinsic limitations of short reads in resolving complex rearrangements necessitated the use of long-read sequencing in these studies (2–6). Long-read sequencing from PacBio HiFi (5) and ONT (4–6) enables reads that span entire breakpoint intervals, unambiguously linking viral and human sequences on single DNA molecules. In addition, Pang et al. integrated PacBio long-read RNA sequencing to identify viral-human fusion transcripts with novel splicing patterns (2). Among these studies (2–5), AmpliconArchitect (74) has emerged as the primary computational framework to reconstruct hybrid ecDNA architecture. AmpliconArchitect detects focal copy number gains, infers structural variants, and constructs breakpoint graphs to reconstruct circular amplicons. For hybrid ecDNA analysis, this tool was adapted to operate on hybrid reference genomes (e.g., GRCh38 + HPV16) and to incorporate viral-specific detection modes (2, 3, 5) for hybrid ecDNA detection. Complementing this graph-based approach, Porter et al. employed *de novo* long-read assembly combined with circularity prediction to identify eight candidate hybrid ecDNA structures across 72 primary cervical tumors (64), demonstrating that assembly-based methods not reliant on focal amplification detection can also recover hybrid ecDNA structures in large patient cohorts. Long-read sequencing coupled with AmpliconArchitect or other newly developed computational tools (75) are expected to further expand the discovery and resolution of hybrid ecDNA structures in HPV-associated cancers and potentially in malignancies driven by other oncogenic viruses.

Beyond sequencing, DNA fluorescence *in situ* hybridization (FISH) on metaphase chromosomes provides orthogonal validation that hybrid structures exist as extrachromosomal entities (1, 3, 5, 8, 76). Two studies used multi-color DNA FISH with probes targeting viral sequences and human genomic sequences—using locus-specific probes (3) or whole chromosome paint probes (5)—to confirm hybrid ecDNA located outside condensed mitotic chromosomes. While DNA FISH provides essential static validation, live-cell imaging has emerged as a powerful approach to study ecDNA behavior in its native nuclear context (18, 20, 22, 23). Live-cell time-lapse microscopy combined with CRISPR-mediated *tetO* array knock-in strategies has revealed that ecDNA dynamically clusters into hubs, hitchhikes on mitotic chromosomes (18, 22), and asymmetrically segregates into daughter cells (23). Another CRISPR-based live-cell ecDNA labeling method, ecTag, has enabled the visualization of the dynamic interaction of ecDNA with transcriptional machinery (20) and with nuclear condensates for rewiring chromatin connectivity (77). Extending these live-cell imaging approaches to hybrid ecDNA would enable real-time tracking of viral-human ecDNA and address fundamental mechanistic questions regarding transcriptional regulation, mitotic segregation, and responses to therapeutic perturbations. Notably, ecTag relies on a single guide RNA targeting ecDNA-specific breakpoint junctions, allowing labeling without the need for CRISPR-mediated insertion of DNA arrays (20, 77). However, ecTag exhibits diminished signal during mitosis, likely due to chromatin condensation, which limits its utility for studying ecDNA segregation mechanisms (20). Consequently, the choice of imaging strategy should be carefully tailored to the specific biological questions being addressed.

## IMPLICATIONS FOR CANCER THERAPY

Hijacking viral episomes onto ecDNA, together with the emergence of *de novo* enhancer architectures, creates unique and targetable dependencies in hybrid ecDNA-positive cancers. These vulnerabilities arise from the convergence of enhancer-driven transcriptional amplification, co-amplification of immune evasion genes and others, host factor-mediated ecDNA maintenance, and heightened DNA damage repair dependencies.

### Enhancer architecture as therapeutic vulnerability

Hybrid ecDNA structures display a recurrent architectural motif in which HPV *E6*/*E7* oncogenes are regulated by *de novo* viral-somatic enhancer complexes (3). Nakagawa et al. performed CRISPRi to interrogate the function of these hybrid enhancers and observed reduced viral oncogene expression and proliferation upon silencing the enhancer directly contacting the *E6*/*E7* promoter, with effects specific to hybrid ecDNA-positive cells (3). Similarly, Tian et al. identified *de novo* enhancers that co-localize with HPV integration loci on hybrid ecDNA and engage in extensive long-range chromatin interactions with chromosomal loci. CRISPR-mediated deletion of the HPV integration region weakened these enhancers and reduced hybrid ecDNA copy number, leading to substantial suppression (up to ~94%) of trans-regulated oncogenic target genes and robust growth inhibition (4). Together, these studies establish enhancer-mediated transcription as a key dependency of hybrid ecDNA tumors, positioning enhancer-targeted therapies as rational and selective therapeutic strategies.

**Fig 2:**
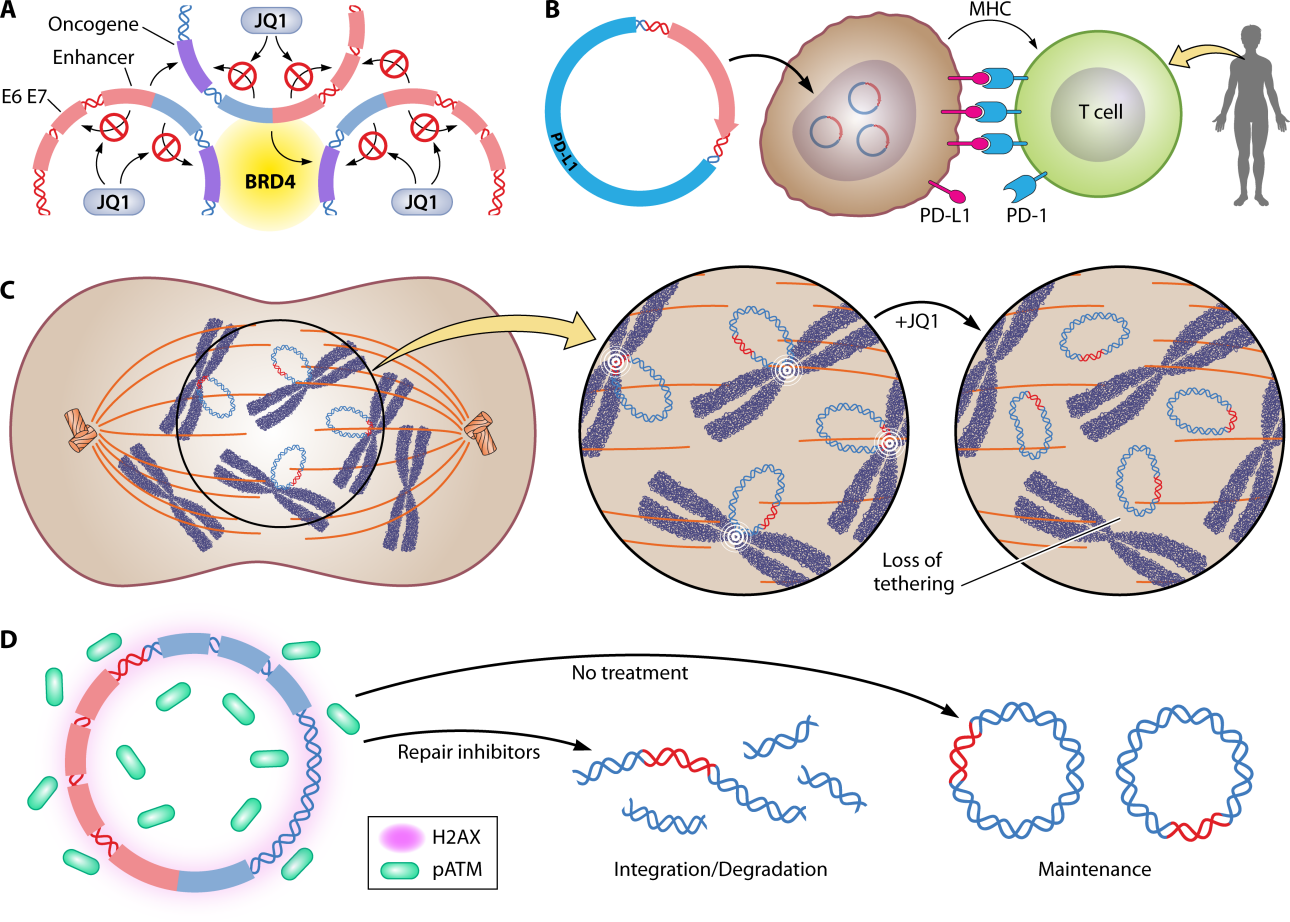
Therapeutic vulnerabilities in hybrid ecDNA-positive cancers. (**A**) Host proteins like BRD4 (yellow condensate) may promote the clustering and expression of hybrid ecDNA molecules (viral = red, human = blue) through inter- and intra-molecular interactions, creating a therapeutic vulnerability exploitable by BET inhibitors. (**B**) Hybrid ecDNA co-amplifies *CD274* (PD-L1, blue) with viral oncogenes (red). PD-L1 engages PD-1 on T cells, suppressing antitumor immunity. (**C**) During mitosis, hybrid ecDNA is thought to hitchhike on condensed chromosomes via tethering mechanisms involving BRD4 or transcription-associated RNA potentiated by BRD4. JQ1 treatment can disrupt this interaction, resulting in ecDNA release and clearance during division. (**D**) Hybrid ecDNA exhibit persistent DNA instability, likely triggering recurrent DNA damage response (γH2AX, pink; pATM, green). Targeting DNA repair machinery may promote hybrid ecDNA loss or aberrant integration.

In conventional ecDNA, enhancer activity is amplified through cooperative hub formation, whereby spatial clustering of multiple ecDNA copies drives exceptionally high transcriptional output (18). Nakagawa et al. observed analogous clustering of hybrid ecDNA by DNA FISH and demonstrated that pharmacologic inhibition of BRD4 with JQ1 reduces hybrid ecDNA copy number, disrupts its spatial organization, and dampens transcriptional activity in HPV-positive oropharyngeal cancers (3), mirroring findings in conventional ecDNA (18). Importantly, this effect is tumor-context specific. JQ1 treatment reduced *E6/E7* expression in a dose- and time-dependent manner exclusively in hybrid ecDNA-positive cell lines (3). Proliferation assays and patient-derived xenograft models confirmed this specificity, with significant growth inhibition observed only in hybrid ecDNA-positive tumors (3). These convergent observations support a model in which BET inhibition perturbs hybrid ecDNA topology and disrupts BRD4-mediated enhancer activity essential for oncogene overexpression (Fig. 2A). This enhancer-centric dependency suggests a therapeutic window exploitable by BET inhibitors.

### Amplification-associated vulnerabilities: PD-L1 co-amplification

Beyond viral oncogenes, hybrid ecDNA can capture and co-amplify nearby host genes, generating a broad set of oncogenic and immune-regulatory liabilities. Individual cases include amplification of classical oncogenes such as *MYC* (5) and *EGFR* (2), as well as immune checkpoint gene *CD274* (encoding programmed death-ligand 1, or PD-L1), which was overexpressed 24.4-fold in one hybrid ecDNA-positive tumor (2). PD-L1 suppresses cytotoxic T cell function by engaging its receptor PD-1 on T cells, promoting an immunosuppressive tumor microenvironment (Fig. 2B) (78). When amplified on hybrid ecDNA, PD-L1 overexpression may create focal immunosuppression that is difficult to overcome with checkpoint inhibitors alone.

While elevated PD-L1 expression may blunt antitumor immunity, immune checkpoint blockade can restore cytotoxic T cell responses in immunosuppressed tumor microenvironments (79). BET inhibition suppresses hybrid ecDNA-driven transcription (3), which may concurrently reduce oncogene and PD-L1 expression, and may transiently sensitize tumor cells. This potentially creates a therapeutic window during which combining BET inhibition and checkpoint blockade could amplify antitumor immunity in hybrid ecDNA-positive tumors. This rational combination exploits the dual oncogenic and immunomodulatory dependencies encoded within hybrid ecDNA.

### Hybrid ecDNA exploitation of BRD4: a potential mechanism for E2-independent mitotic tethering

Hybrid ecDNA appears to exhibit a distinct relationship with the viral E2 protein compared to both episomal and chromosomally integrated forms. In cell lines harboring a predominance of virus-host concatemers in intrachromosomal DNA, Akagi et al. observed deletions in *E1* and/or *E2* with sustained high *E6*/*E7* expression, consistent with classical integration-driven derepression (5). Notably, heterocateny, including hybrid ecDNA forms, was also detected in samples lacking detectable *E1* and *E2* expression (tumor 5, GUMC-395, and VU147) (5), indicating that canonical *E1*/*E2*-dependent viral replication machinery may not be required for hybrid ecDNA formation or maintenance. Pang et al. similarly observed loss of *E1/E2* expression in hybrid ecDNA tumors alongside elevated *E6/E7* (2). In contrast, four of the five primary tumors with predominantly ecDNA-form concatemers retained *E1* and *E2* expression yet exhibited comparably high *E6*/*E7* levels (5), indicating that high *E6*/*E7* expression can occur even when the E2 repressor is intact—a finding that complicates the classical integration-driven derepression model. These discrepancies may partially reflect methodological differences, including sequencing approach (short-read versus long-read) and resolution (bulk versus individual virus–host concatemer). Together, these limited observations indicate that hybrid ecDNA can sustain high *E6*/*E7* expression independently of *E2* status. Furthermore, the persistence of heterocateny and hybrid ecDNA in *E1*/*E2*-deficient samples suggests that canonical viral maintenance machinery may not be required, pointing to alternative mechanisms governing the replication and mitotic inheritance of hybrid ecDNA.

It is tempting to speculate that hybrid ecDNA co-opts the BRD4-dependent mitotic tethering mechanism previously described for conventional ecDNA (18, 22). Hybrid ecDNA is enriched in highly acetylated chromatin (3), a histone mark known to recruit BRD4 through its bromodomains (80). Such acetylation-rich regions at enhancers/promoters could provide a multivalent binding platform for BRD4 (80), enabling chromatin association of hybrid ecDNA with mitotic chromosomes. Prior studies have also shown that RNA-mediated interactions can stabilize ecDNA-chromosome associations during mitosis (22). Because BRD4 is also a potent transcriptional coactivator, BRD4-driven transcriptional activity may alternatively reinforce hybrid ecDNA inheritance through RNA-mediated tethering mechanisms (22). Whether BRD4 mediates hybrid ecDNA segregation primarily through biophysical interactions (80), transcription-associated RNA scaffolding (22), a combination of both, or via other mechanisms remains an important open question. If validated, this dependency would extend the therapeutic impact of BET inhibition beyond transcriptional suppression to disruption of hybrid ecDNA inheritance itself, potentially leading to progressive ecDNA loss across successive cell divisions (Fig. 2C).

### DNA repair pathway dependencies

Pervasive transcription across the circular topology of conventional ecDNA induces transcription-replication conflict and replication stress-associated DNA damage, creating a dependency on the S-phase checkpoint kinase CHK1 for ecDNA maintenance (81). CHK1 inhibition leads to the accumulation of unrepaired DNA damage and tumor cell death (81), and CHK1 inhibitors are currently being evaluated in clinical trials for ecDNA-amplified tumors (clinical trial number NCT05827614). In parallel, another study demonstrates that ecDNA replication and maintenance require DNA damage response, with repair of double-strand breaks that persist into mitosis relying primarily on the alternative non-homologous end joining (alt-NHEJ) pathway; inhibition of key alt-NHEJ components leads to substantial ecDNA loss (82). By analogy, hybrid viral-human ecDNA may impose similar DNA repair dependencies (81, 82). The dynamic instability arising from repeated excision/insertion cycles (5), elevated transcription-replication conflicts contributed by viral/human promoters and hybrid enhancers, and complex replication fork dynamics across chimeric sequences are expected to generate persistent DNA damage, necessitating continued reliance on host DNA repair machinery for hybrid ecDNA maintenance. Consequently, hybrid ecDNA-positive tumors may exhibit elevated sensitivity to inhibitors targeting these DNA repair pathways (Fig. 2D).

## CONCLUSION

The discovery of hybrid viral-human ecDNA reveals a previously unrecognized convergence between viral oncogenesis and extrachromosomal gene amplification. Viral integration does not merely insert viral oncogenes into host chromosomes; instead, it can catalyze ecDNA formation, generating circular chimeric DNA structures that fuse viral oncogenes with captured human genomic sequences and give rise to *de novo* regulatory elements absent from either chromosomal DNA or canonical viral episomes. This convergence endows hybrid ecDNA with a distinctive set of molecular features as follows: (i) *de novo* composite enhancer formation, (ii) cooperative activation of viral and co-amplified human genes, and (iii) a capacity to exert gained *trans*-regulatory effects across host chromosomes.

Despite these insights, significant knowledge gaps remain. First, does the apparent restriction of hybrid ecDNA to HPV-associated cancers, despite extensive study of cancers driven by other oncogenic viruses (e.g., EBV and HBV), reflect biological constraints unique to HPV or limitations in detection methods? Second, fundamental mechanistic questions regarding hybrid ecDNA biogenesis, maintenance, and functional contribution to cancer progression remain incompletely understood. Third, the evolutionary and clinical significance of hybrid ecDNA remains unclear: do these structures confer a selective advantage or arise through largely neutral processes, and can they be exploited as new prognostic biomarkers in HPV-associated cancers?

Importantly, hybrid ecDNA may encode mechanistically distinct vulnerabilities that can be therapeutically exploited. BET inhibition could disrupt multiple layers of hybrid ecDNA dependence simultaneously: perturbing BRD4-mediated transcriptional hubs and hybrid ecDNA spatial clustering, suppressing the expression of co-amplified oncogenic and immune evasion genes, and potentially impairing mitotic tethering to promote progressive hybrid ecDNA loss. In parallel, the heterocateny-based model of hybrid ecDNA formation—characterized by dynamic cycles of chromosomal insertion, excision, and recombination of concatemerized viral and host DNA—would be expected to impose chronic DNA damage stress and create dependencies on DNA damage responses. Such a model predicts that hybrid ecDNA-positive tumors may exhibit heightened sensitivity to DNA repair pathway inhibitors, revealing orthogonal and combinatorial vulnerabilities intrinsic to the hybrid structure itself.

Preclinical validation in additional patient-derived xenograft models will be essential to evaluate these hybrid ecDNA-directed therapeutic strategies for both efficacy and durability of response. Ultimately, clinical trials in HPV-associated malignancies will determine whether the molecular dependencies encoded within hybrid ecDNA can be therapeutically exploited. If successful, the study of hybrid ecDNA may reveal not merely a novel mechanism of viral oncogenesis, but an inadvertent vulnerability: an Achilles’ heel forged by the very genomic instability that drives tumor evolution.
